# Sexually Transmitted Infections in 2000–2018 in a Specialised Centre: Comparison between Pre-Crisis, Crisis, and Post-Crisis Period

**DOI:** 10.3390/jcm12165254

**Published:** 2023-08-12

**Authors:** María Sánchez-Torres, Beatriz Espadafor-López, Isabel Llavero-Molino, María Adelaida Álvarez-Serrano, Inmaculada García-García, César Hueso-Montoro, María Ángeles Pérez-Morente

**Affiliations:** 1 Virgen de la Arrixaca University Clinical Hospital, El Palmar, 30120 Murcia, Spain; marikillast@gmail.com; 2 Virgen de las Nieves University Hospital, Center for Sexually Transmitted Infections, Andalusian Health Service, 18012 Granada, Spain; beaespadafor@gmail.com; 3 Maternal and Child Hospital, 23007 Jaén, Spain; isa_llavero_13@hotmail.com; 4 Department of Nursing, Faculty of Health Sciences, University of Granada, 51001 Ceuta, Spain; 5 Department of Nursing, Faculty of Health Sciences, University of Granada, 18071 Granada, Spain; igarcia@ugr.es; 6 Department of Nursing, Faculty of Health Sciences, University of Jaén, 23071 Jaén, Spain; mmorente@ujaen.es; 7 Instituto Biosanitario Granada (IBS. Granada), 18012 Granada, Spain; 8 Centro de Investigación Mente, Cerebro y Comportamiento (CIMCYC) of the University of Granada, 18011 Granada, Spain

**Keywords:** sexually transmitted diseases, risk factors, social determinants of health, economic recession, sexual behaviour, epidemiology

## Abstract

(1) Background: Sexually Transmitted Infections (STIs) are a major public health problem due to their consequences in sexual and reproductive health. There is a close link between the crisis and the increase in communicable diseases. The objective of this study was to analyse the evolution of Sexually Transmitted Infections during the period 2000–2018 in the population attending the Centre for Sexually Transmitted Diseases and Sexual Orientation in Granada (Spain), specifically comparing the pre-crisis, crisis, and post-crisis periods. (2) Methods: A retrospective, observational, and analytical study was conducted by reviewing medical records. The sample analysed comprised 1666 cases. (3) Results: During the pre-crisis period (2000–2007), the percentage of diagnoses was 41.6% (*n* = 126) compared to 58.4% (*n* = 177) of negative results; during the crisis, the percentages were 63.5% (*n* = 183) and 36.5% (*n* = 105), respectively; and during the post-crisis period, the percentages were 42.9% (*n* = 157) and 57.1% (*n* = 209), respectively. The variables that were significantly associated with STI diagnosis were the time periods analysed, sexual orientation, occupation, and age at first intercourse. The evolution of the number of positive diagnoses during the entire study period showed a trend of progressive increase in Sexually Transmitted Infections from 2000 to 2018. (4) Conclusions: The period of economic crisis presented a higher risk of infection, although this is a finding with certain limitations due to the lack of homogeneity between the periods analysed.

## 1. Introduction

Sexually Transmitted Infections (STIs) are a major public health problem, with more than one million people contracting them each year and their consequences leading to sexual and reproductive health complications [1]. This results in increased expenditure and, therefore, increased consumption and use of health and social care services [2]. This situation could further compromise the sexual and reproductive health of the most vulnerable population groups and those with the highest rates of STIs, such as sex workers, men who have sex with men, injection drug users, prisoners, nomadic populations, and adolescents [1].

Globally, some 374 million people are infected with syphilis, chlamydia, trichomoniasis, and blennorrhoea each year, while herpes simplex virus (HSV) affects an estimated 500 million people aged 15–49 years [1]. In Europe, the most frequent cause of morbidity is STIs, with over 400,000 cases of chlamydia, 89,000 cases of gonorrhoea, and 33,000 cases of syphilis diagnosed in 2017, according to the European Surveillance Authority [3]. In 2018 alone, in Spain there were 5079 cases of syphilis, 13,109 of Chlamydia trachomatis, 11,044 of gonococcal infection, and 282 of Lymphogranuloma venereum. These figures show a progressive increase in rates, more frequent in men than in women, with the exception of Chlamydia trachomatis, which is mostly contracted by women under 25 years of age [4].

The transmission of infectious agents has been increasing both for contextual reasons in the work and home environment, as well as for behavioural reasons such as drug use, unsafe sexual practices, and lack of therapeutic and preventive measures [5]. With economic crises, studies describe a worsening of some health indicators, especially in the most affected countries and in particularly vulnerable groups such as the unemployed, low-income earners, immigrants, and ethnic minorities [6], and at critical ages such as childhood, youth, and old age [5,7]. There is a close link between the crisis and the increase in communicable diseases due to the deterioration in epidemic prevention and control programs, poor living conditions, and the worsening in socioeconomic inequalities characterised by the destruction of employment, and by work and precarious housing. [6]. In addition to the above, the growing difficulty in accessing social and health services, and insufficient resources for epidemiological surveillance and public health systems due to abusive cuts, should be noted [5].

The austerity measures imposed in the crisis years include increased privatisation in the management of public health services, wage cuts, and job insecurity, together with cuts in the service sector and an increase in the level of unemployment, all of which contribute to this increase in communicable diseases and, consequently, in STIs [5]. Low living standards in times of crisis increase economic pressure on many households, leading to overcrowding, malnutrition, and reduced immunity due to high levels of stress. As a consequence, infectious diseases have negative effects, including economic ones, making recovery even more difficult [2]. In Spain, between 2008 and 2014, coinciding with the period of crisis, some STIs that were thought to have been suppressed, such as gonorrhoea and syphilis, reappeared [8], together with an increase in the incidence of other infections such as hepatitis, HIV, and Human Papilloma Virus (HPV), when it was believed that there was detailed control of the latter [9].

According to the conclusions of the document on “Epidemiological surveillance of STIs in Spain in 2018”, the increasing trend of gonococcal infection and syphilis, observed since the beginning of the 2000s, continues [4]. The present study is a continuation of a previously published study [10] which analysed the evolution and behaviour of STIs, from the period before and during the economic crisis, in users attending a specialised centre in the city of Granada, Spain. The analysis in the current research includes the period after the economic crisis, based on the premise that improvements in socioeconomic conditions after the recession years could have led to a decrease in the number of STI diagnoses. This research therefore provides evidence on how the period after the economic crisis has influenced the evolution of STIs. Based on the above, the aim of this study was to analyse the evolution of STIs during the period 2000–2018 in the population attending the Centre for Sexually Transmitted Diseases in the province of Granada, specifically comparing the pre-crisis, crisis, and post-crisis periods.

## 2. Materials and Methods

### 2.1. Design and Data

A retrospective, observational, and analytical study was conducted by reviewing medical records from the Centre for Sexually Transmitted Diseases and Sexual Orientation in Granada (Spain). This is a reference centre in southern Spain.

Data were obtained from the records of adult subjects without cognitive impairment (ascertained by accessing the clinical history) who came to the centre for consultation associated with the presence or suspected transmission of an STI. The clinical history included four options (symptomatology, control, contact tracing, and HIV) and those in which one of these options was ticked were selected. Data from sex workers were excluded because of the potential bias that could result from the inclusion of this group, which is more exposed to STIs.

### 2.2. Data Collection

The study period covered the years 2000 to 2018. Additionally, the periods 2000–2007, 2008–2014, and 2015–2018 were compared. These periods differ according to the impact of the economic crisis during the second period. Therefore, the pre-crisis period was considered as 2000–2007, the crisis period as 2008–2014, and the post-crisis period as 2015–2018. Subsequent periods have not been included in this research as the pandemic prevented data collection, an issue that will be addressed in future research.

The sample was drawn from the total number of records included in that period, in this case 26,834 records. To determine the sample size, a finite population was considered, with a 95% confidence level, a precision of 3%, and an expected proportion of STIs of 50% (to maximise the sample size), obtaining an *n* equal to 1026 records. Simple random sampling without replacement was used. The method of sample attainment was based on new records per year, from which the first and last record number of that year was taken, thus obtaining a proportional sample per year. When the selected medical record did not meet the inclusion criteria, the immediately preceding medical record was selected; if it also did not meet the criteria, the medical record immediately following the initial one was selected. If the inclusion criteria were not met in both cases, the selection continued backwards and forwards until a medical record that met the criteria was obtained.

An ad hoc data collection sheet was prepared based on the study variables. Subsequently, a computerised database was designed to contain the information compatible with the statistical analysis software used. Data collection was carried out face-to-face at the centre using paper medical records by three researchers who were previously trained to ensure a homogeneous and consensual process. An initial pilot study was conducted on 110 medical record to refine the data collection sheet and clarify doubts about some variables. The people who participated in the data collection had university training in health sciences.

### 2.3. Variable Selection

The dependent variable was STI diagnosis, which was coded as a dichotomous variable (Yes/No). Histories in which the positive or negative result of the STI diagnosis was explicitly recorded according to medical criteria were considered [11]. The remaining variables were grouped into two categories: socio-demographic characteristics and risk indicators. The data for each variable were collected as they appeared on the record sheet used in the centre where the research was carried out, adapting the categories of some of them to facilitate the analysis.

### 2.4. Data Analysis

For the statistical analysis, descriptive statistics were used to obtain the median (Me) and the interquartile range (IQR) for continuous variables. For categorical variables, absolute frequency (*n*) and percentage (%) were used. To analyse the normality of the continuous variables, the values of skewness and kurtosis were analysed, as well as the Kolmogorov–Smirnoff test, and a non-normal distribution was observed.

Subsequently, non-parametric hypothesis testing was carried out. The Kruskal–Wallis and U Mann–Whitney tests were used. To compare categorical variables, contingency tables were created and the chi-square test (χ^2^) was performed; when this could not be applied, the generalisation of Fisher’s exact test was used.

To complete the analysis, a multiple logistic regression analysis was performed, taking STI diagnosis as the outcome variable. In the selection of covariates, we first included those that were significantly associated with the main variable in the bivariate analysis, and then excluded those that lost statistical significance in the regression analysis. For each variable included in the model, the odds ratio (OR) was calculated with the 95% confidence interval (CI). Once the model was generated, the fit conditions were checked. Collinearity between variables was investigated by calculating the variance inflation factor (VIF); linearity of the dependent variable with the continuous variables included in the model was checked, and calibration was determined using the Hosmer–Lemeshow goodness-of-fit test. Finally, discrimination was determined from the value of the area under the Receiver Operating Characteristic (ROC) curve. The number of records selected ensured a sufficient number of cases per variable entered in the model, according to the method described by Peduzzi et al. [12].

In all analyses, a value of *p* < 0.05 was considered statistically significant. Calculations were performed with R commander software, R version 3.2.2 (https://www.r-project.org/, R-UCA Project, http://knuth.uca.es/R, accessed on 15 January 2023) and IBM SPSS© v.26 (IBM Corporation, Armonk, NY, USA) using the corporate licence of the University of Granada.

### 2.5. Ethical Considerations

This study was approved by the Biomedical Research Ethics Committee of the province of Granada.

## 3. Results

The final sample consisted of 1666 medical records, of which 656 were from the pre-crisis period, 629 from the crisis period, and 381 from the post-crisis period. The results show that in all three periods, the populations were homogeneous in terms of age, sex, and marital status, with statistically significant differences found by nationality, educational level, and sexual orientation (Table 1).

In terms of risk indicators, there were significant differences between the three periods in age at first intercourse, time since last unprotected sex, partners in the last month and in the last year, lifetime partners, regular partner, and contact with sex workers. The population was homogeneous for all other variables (Table 2).

**Figure 1:**
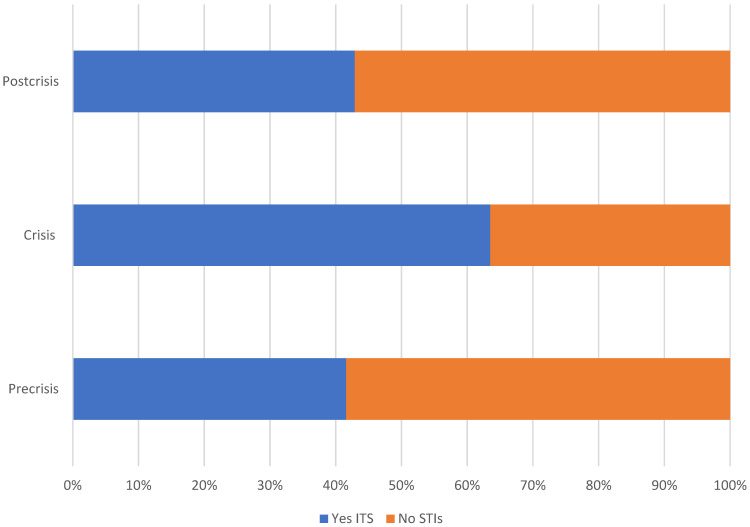
Proportion of STIs (Yes/No) in all three study periods.

When analysing the presence of STIs between the three periods, 957 medical records were analysed: 303 from the non-crisis period, 288 from the crisis period, and 366 from the post-crisis period. It was observed that, during the pre-crisis period, the percentage of diagnoses was 41.6% (*n* = 126) compared to 58.4% (*n* = 177) of negative results for STIs; during the crisis, the percentages were 63.5% (*n* = 183) and 36.5% (*n* = 105), respectively; and during the post-crisis period, the percentages were 42.9% (*n* = 157) and 57.1% (*n* = 209), respectively. According to bivariate analysis, this difference was statistically significant (*p* < 0.001) (Figure 1).

**Figure 2:**
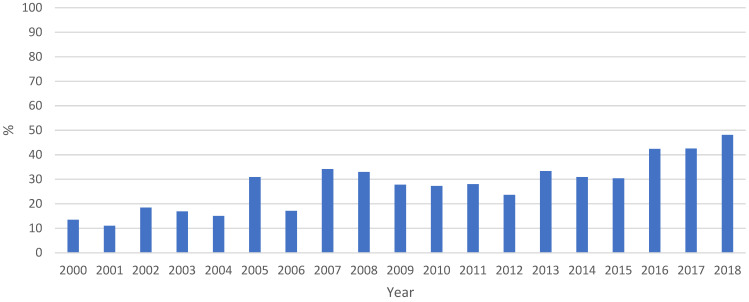
Evolution of the number of STIs over the entire study period. Proportion of STI diagnoses as a function of the total number of cases per year.

If we analyse the evolution of the number of diagnoses throughout the study period, we observe a trend of progressive increase from 2000 to 2018 (Figure 2).

According to the bivariate analysis, the STI variable was also associated with age at first sexual intercourse, sex, nationality, occupation, educational level, and sexual behaviour (Table 3).

The regression model generated is shown in Table 4. The variables included that were significantly associated with STI diagnosis were the time periods analysed, sexual orientation, occupation, and age at first intercourse.

## 4. Discussion

This study provides data on the evolution of STIs and their behaviour during the period 2000–2018 in a population that attended a specialised reference centre for the care of these infections, comparing three different periods (pre-crisis, crisis, and post-crisis) due to the economic crisis that occurred between 2008 and 2014.

The profile of the population analysed and delimited in the three periods significantly show a progressive decrease in the proportion of the immigrant population attending the centre, which was accompanied by an increase in the population with a higher level of education. These findings coincide with the official data published by the National Institute of Statistics [13] on the foreign population in Spain from 1998 to 2021 and with the study by the EPI-VIH group [14], as well as with those published by the INE [15] regarding the progressive increase in the educational level of the Spanish population during the post-crisis period. Another striking fact is the progressive increase in the proportion of subjects with homosexual or bisexual orientation compared to those with heterosexual orientation. If we analyse the risk factors, it is worth highlighting the increase in the time of unprotected sexual relations in the post-crisis period compared to the crisis period, as well as an increase in the number of partners.

In relation to the behaviour of STIs, it is worth recalling that the study preceding this research [10] concluded that the probability of infection was higher in the crisis period compared to the pre-crisis period. In the new period analysed, which was characterised by economic recovery, there was a lower proportion of positive diagnoses compared to the crisis period, which was similar to the pre-crisis period. These findings are in line with what has already been published on how economic crises affect the health of the population and, in particular, infectious diseases such as STIs [2,10,16,17,18,19].

However, the above hypothesis has nuances that should be clarified in the light of the results obtained. The data on the evolution of STIs in the period of time analysed shows a trend of progressive increase in the number of infections from 2000 to 2018, so that, in absolute terms, the number of STIs was higher in recent years than in previous years. This coincides with the epidemiological data for Spain in recent years, which maintain an upward trend in the diagnosis of these infections; this was especially marked from 2005 to 2018, both in gonorrhoea and syphilis [20], as well as in Chlamydia trachomatis, with more than double the number of notifications in 2019 compared to 2016 [21]. Although the evolution of the main STIs in Spain follows the same upward pattern as in Europe, Spain is one of the countries with the highest rates [22].

It is important to note that the number of medical records with a positive or negative result of the clinical diagnosis recorded in the centre where the research was carried out was higher in the post-crisis period, despite fewer years of analysis. This, on the one hand, shows an improvement in screening procedures due to greater control of the diagnostic procedure, which could be due to improved economic conditions and greater accessibility to the health system following the restrictive measures of Royal Decree Law 16/2012 [23]. On the other hand, these data must be taken into account to contextualise the difference observed in terms of the proportion of STIs in the three periods analysed, which could call into question the attribution to the economic crisis of a greater probability of infection of these infections.

Finally, regression analysis also shows that starting sex earlier, being gay, and working (not being a student) are factors that predict a higher likelihood of STI transmission. Evidence confirms that early sexual debut is a contributing factor to an increase in STIs [24,25]. Behavioural patterns in the adolescent population confirm that young people are starting to engage in full sexual relations at an increasingly younger age [26], which, together with a lack of information and guidance, and the immaturity at younger ages, exposes young people to significant risks [27]. Hence, this population group is considered among the most vulnerable to STIs [1,28,29]. Scientific evidence also places homosexual men and other men who have sex with men (MSM) [30] among the groups vulnerable to infection by these infections, which is in line with the results of this research. Likewise, the relationship found between being in employment and having a higher probability of STIs could be justified because a better economic situation may predispose to risk behaviour. In this sense, a previous study found an association between socioeconomic status and the prevalence of Chlamydia trachomatis in three regions with different levels of development, observing higher prevalence in the most economically developed region; among the reasons given by the authors was the greater likelihood of extramarital sexual behaviour and multiple sexual partners [31].

This study has some limitations, one of which is related to the high percentage of missing values in some variables, which is a common handicap when researching medical records. Furthermore, it is important to be cautious with the extrapolation of results, as this was a single-centre, single-province study, which limits its external validity. Nevertheless, the WHO has emphasised in recent years the need for local-level data to improve the approach to STIs [32]. It should also be borne in mind that, despite having analysed a large time series and having a high number of cases, as this was a cross-sectional design, the associations found should be considered to be causal hypotheses that should be verified with other studies. Finally, the post-crisis period has fewer years of analysis due to the outbreak of the COVID19 pandemic, which poses the challenge of assessing its influence on the behaviour of STIs in future research.

## 5. Conclusions

In conclusion, the results show that the period of economic crisis, in proportional terms, presented a higher risk in the study population with respect to STI infection. However, there was a clear trend towards a progressive increase in these infections from 2000 to 2018, which could be explained by the improvement in screening systems. This could raise doubts about the effect of the economic crisis on these infections, at least in the series analysed.

Our results also confirm the existence of already studied risk factors such as early sexual debut or the vulnerability of certain groups. It is therefore a priority objective to pay attention to risk behaviours and vulnerable groups in sexual health prevention programmes. In addition, the social and economic context of the country should be taken into account, so that in situations of recession the health system should be strengthened to minimise its impact on the health of the population.

## Figures and Tables

**Table 1:** Socio-demographic characteristics.

Variable	Pre-Crisis (*n* = 656)			Crisis *(*n** = 629)			Post-Crisis (*n* = 381)			*p* ^c^
	*n*	Me ^a^	IQR ^b^	*n*	Me ^a^	IQR ^b^	*n*	Me ^a^	IQR ^b^	
**Age (*n* = 1664)**	656	26	9	629	26	9	379	26	13	0.958
**Variables**	***n***		**%**	***n***		**%**	***n***	**%**		***p*** **^d^**
**Sex (*n* = 1666)**										
Man	375		57.2%	392		62.3%	225	59.1%		0.166
Woman	281		42.8%	237		37.7%	156	40.9%		
**Nationality (*n* = 1649)**										
Spanish	510		79.4%	522		83.3%	330	86.8%		0.009
Another	132		20.6%	105		16.7%	50	13.2%		
**Occupation (*n* = 1551)**										
Another	307		53.0%	353		57.5%	198	55.3%		0.300
Student	272		47.0%	261		42.5%	160	44.7%		
**Level of education (*n* = 1584)**										
Superiors	315		51.6%	351		57.9%	217	59.0%		0.032
Other	295		48.4%	255		42.1%	151	41.0%		
**Marital status (*n* = 1654)**										
Single	526		80.7%	529		84.5%	319	84.8%		0.110
Another	126		19.3%	97		15.5%	57	15.2%		
**Sexual orientation (*n* = 1634)**										
Straight	564		87.7%	460		74.7%	276	73.6%		<0.001
Bisexual	16		2.5%	34		5.5%	27	7.2%		
Homosexual	63		9.8%	122		19.8%	72	19.2%		

^a^ Median; ^b^ Interquartile Range; ^c^ Kruskal–Wallis test; ^d^ chi-square test (χ^2^).

**Table 2:** Risk indicators.

Variable	Pre-Crisis (*n* = 656)			CRISIS (*n* = 629)			Post-Crisis (*n* = 381)			*p* ^c^
	*n*	Me ^a^	IQR ^b^	*n*	Me ^a^	IQR ^b^	*n*	Me ^a^	IQR ^b^	
**Age at first sexual intercourse (*n* = 1081)**	288	18	3	449	17	2	344	17	3	<0.001
**Variables**	***n***		**%**	***n***		**%**	***n***		**%**	***p*** **^d^**
**Last unprotected sexual contact (*n* = 1071)**										
Never	6		1.5%	38		8.4%	10		4.4%	<0.001
Less than 1 month	155		39.7%	189		41.8%	100		43.7%	
Between 1 and 6 months	169		43.3%	183		40.5%	92		40.2%	
Between 6 and 12 months	21		5.4%	24		5.3%	15		6.6%	
More than 12 months	39		10.0%	18		4.0%	12		5.2%	
**Partners in the last month (*n* = 1568)**										
0–1	501		85.2%	518		84.2%	241		66.0%	<0.001
2	56		9.5%	54		8.8%	78		21.4%	
3–5	28		4.8%	34		5.5%	34		9.3%	
More than 5	3		0.5%	9		1.5%	12		3.3%	
**Couples in the last year (*n* = 1551)**										
0–1	224		38.6%	219		36.1%	90		24.8%	<0.001
2	139		23.9%	122		20.1%	69		19.0%	
3–5	139		23.9%	158		26.0%	105		28.9%	
6–10	53		9.1%	80		13.2%	55		15.2%	
11–20	16		2.8%	15		2.5%	30		8.3%	
More than 20	10		1.7%	13		2.1%	14		3.9%	
**Lifetime partners (*n* = 537)**										
0–10	79		61.2%	108		51.2%	94		47.7%	0.034
10–20	24		18.6%	51		24.2%	37		18.8%	
More than 20	26		20.2%	52		24.6%	66		33.5%	
**Stable couple (*n* = 1561)**										
Yes	401		67.3%	376		61.8%	198		55.5%	0.001
No	195		32.7%	232		38.2%	159		44.5%	
**Relationships with sex workers (*n* = 770)**										
Yes	75		27.4%	53		18.3%	39		18.8%	0.017
No	199		72.6%	236		81.7%	168		81.2%	
**Regular partner with STI symptoms (*n* = 577)**										
Yes	66		38.8%	108		42.9%	56		36.1%	0.383
No	104		61.2%	144		57.1%	99		63.9%	
**Drug use (*n* = 1001)**										
Yes	108		37.0%	128		34.8%	98		28.7%	0.069
No	184		63.0%	240		65.2%	243		71.3%	
**Previous STIs (*n* = 1397)**										
Yes	447		80.0%	382		78.6%	265		75.3%	0.244
No	112		20.0%	104		21.4%	87		24.7%	

^a^ Median; ^b^ Interquartile Range; ^c^ Kruskal–Wallis test; ^d^ chi-square test (χ^2^).

**Table 3:** STIs vs. other variables.

	No STIs (*n* = 491)			Yes ITS (*n* = 466)			*p* ^c^
Variables	*n*	Me ^a^	IQR ^b^	*n*	Me ^a^	IQR ^b^	
**Age (*n* = 956)**	490	26	12	466	26	11	0.614
**Age at first sexual intercourse (*n* = 593)**	314	17	3	279	17	3	<0.001
**Variables**	**n**		**%**	**n**		**%**	***p*** **^d^**
**Sex (*n* = 957)**							
Man	291		59.3%	315		67.6%	0.008
Woman	200		40.7%	151		32.4%	
**Nationality (*n* = 950)**							
Spanish	408		83.8%	411		88.8%	0.026
Another	79		16.2%	52		11.2%	
**Occupation (*n* = 887)**							
Another	227		50.9%	277		62.8%	<0.001
Student	219		49.1%	164		37.2%	
**Level of education (*n* = 914)**							
Top	275		58.6%	229		51.5%	0.029
Another	194		41.4%	216		48.5%	
**Marital status (*n* = 951)**							
Single	395		81.3%	381		81.9%	0.793
Another	91		18.7%	84		18.1%	
**Sexual orientation (*n* = 933)**							
Straight	388		81.2%	342		75.2%	<0.001
Bisexual	34		7.1%	18		4.0%	
Homosexual	56		11.7%	95		20.9%	
**Last unprotected sexual contact (*n* = 549)**							
Never	14		5.3%	17		6.0%	0.950
Less than 1 month	121		45.5%	125		44.2%	
Between 1 and 6 months	108		40.6%	112		39.6%	
Between 6 and 12 months	10		3.8%	14		4.9%	
More than 12 months	13		4.9%	15		5.3%	
**Partners in the last month (*n* = 901)**							
0–1	354		77.5%	342		77.0%	0.427
2	70		15.3%	58		13.1%	
3–5	27		5.9%	35		7.9%	
More than 5	6		1.3%	9		2.0%	
**Partners in the last year (*n* = 897)**							
0–1	157		34.6%	160		36.1%	0.284
2	88		19.4%	76		17.2%	
3–5	119		26.2%	113		25.5%	
6–10	66		14.5%	57		12.9%	
11–20	19		4.2%	23		5.2%	
More than 20	5		1.1%	14		3.2%	
**Couples throughout life (*n* = 317)**							
0–10	84		53.8%	70		43.5%	0.101
10–20	33		21.2%	34		21.1%	
More than 20	39		25.0%	57		35.4%	
**Stable couple (*n* = 898)**							
Yes	290		63.3%	284		64.5%	0.702
No	168		36.7%	156		35.5%	
**Relationships with sex workers (*n* = 465)**							
Yes	52		23.3%	41		16.9%	0.086
No	171		76.7%	201		83.1%	
**Regular partner with STI symptoms (*n* = 371)**							
Yes	69		39.2%	87		44.6%	0.292
No	107		60.8%	108		55.4%	
**Drug use (*n* = 571)**							
Yes	85		28.3%	90		33.2%	0.207
No	215		71.7%	181		66.8%	
**Previous STIs (*n* = 814)**							
Yes	323		78.2%	292		72.8%	0.074
No	90		21.8%	109		27.2%	

^a^ Median; ^b^ Interquartile Range; ^c^ U Mann–Whitney test; ^d^ chi-square test (χ^2^).

**Table 4:** Logistic regression model of variables associated with STIs ^a^.

Variable	OR ^b^ [95% CI] ^c^	*p*	VIF ^d^
**Period**			1.06
Crisis	Ref.	Ref.	
No Crisis	0.55 (0.31–0.94)	0.031	
Post-crisis	0.40 (0.26–0.60)	<0.001	
**Sexual orientation**			1.05
Straight	Ref.	Ref.	
Bisexual	0.35 (0.14–0.76)	0.012	
Homosexual	1.87 (1.17–3.01)	0.009	
**Profession**			1.02
Other	Ref.	Ref.	
Student	0.54 (0.37–0.77)	<0.001	
**Age of first sexual intercourse**	0.88 (0.82–0.95)	0.001	1.08

^a^ Calibration (Hosmer–Lemeshow goodness-of-fit test): X-squared = 4.61, degrees of freedom = 8, *p* = 0.798. Area under the ROC curve = 0.68 (95% CI = 0.63–0.72). *n* = 554. ^b^ Odds ratio (OR); ^c^ 95% Confidence interval (95% CI); ^d^ Variance Inflation Factor (VIF).

## Data Availability

The data that support the findings of this study are available from the corresponding author, upon reasonable request.
